# Accessibility of basic paediatric emergency care in Malawi: analysis of a national facility census

**DOI:** 10.1186/s12889-020-09043-3

**Published:** 2020-06-24

**Authors:** Emily White Johansson, Cecilia Lindsjö, Daniel J. Weiss, Humphreys Nsona, Katarina Ekholm Selling, Norman Lufesi, Helena Hildenwall

**Affiliations:** 1 Department of Women’s and Children’s Health, International Maternal and Child Health, Uppsala University, Akademiska Sjukhuset, SE-751 85 Uppsala, Sweden; 2 Department of Public Health Sciences, Global Health – Health System and Policy Research Group, Karolinska Institutet, SE-171 77 Stockholm, Sweden; 3 Oxford Big Data Institute, Li Ka Shing Centre for Health Information and Discovery, Nuffield Department of Medicine, University of Oxford, Oxford, OX3 7LF UK; 4 Ministry of Health, Integrated Management of Childhood Illness (IMCI) Unit, Lilongwe, Malawi; 5 Ministry of Health, Community Health Sciences Unit, Lilongwe, Malawi

**Keywords:** Malawi, Emergency care, Paediatrics, Health systems

## Abstract

**Background:**

Emergency care is among the weakest parts of health systems in low-income countries with both quality and accessibility constraints. Previous studies estimated accessibility to surgical or emergency care based on population travel times to nearest hospital with no assessment of hospital readiness to provide such care. We analysed a Malawi national facility census with comprehensive inventory audits and geocoded facility locations to identify hospitals equipped to provide basic paediatric emergency care with estimated travel times to these hospitals from non-equipped facilities and in relation to Malawi’s population distribution.

**Methods:**

We analysed a Malawi national facility census in 2013–2014 to identify hospitals equipped to manage critically ill children according to an extended version of WHO Emergency Triage, Assessment and Treatment (ETAT) guidelines. These guidelines include 25 components including staff, transport, equipment, diagnostics, medications, fluids, feeds and consumables that defined an emergency-equipped hospital in our study. We estimated travel times to emergency-equipped hospitals from non-equipped facilities and relative to population distributions using geocoded facility locations and an established accessibility mapping approach using global road network datasets from OpenStreetMap and Google.

**Results:**

Four (3.5, 95% CI: 1.3–8.9) of 116 Malawi hospitals were emergency-equipped. Least available items were nasogastric tubes in 34.5% of hospitals (95% CI: 26.4–43.6), blood typing services (40.4, 95% CI: 31.9–49.6), micro nebulizers (50.9, 95% CI: 41.9–60.0), and radiology (54.2, 95% CI: 45.1–63.0). Nationally, the median travel time from non-equipped facilities to the nearest emergency-equipped hospital was 73 min (95% CI: 67–77) ranging 1–507 min. Approximately one-quarter (27%) of Malawians lived over 120 min from an emergency-equipped hospital with significantly better accessibility in Central than North and South regions (16% vs. 38 and 35%, *p* < 0.001).

**Conclusions:**

There are unacceptable deficiencies in accessibility of basic paediatric emergency care in Malawi. Reliable supply chains for essential drugs and commodities are needed, particularly nasogastric tubes, asthma drugs and blood, along with improved capacity for time-sensitive referral. Further child mortality reductions will require substantial investments to expand basic paediatric emergency care into all Malawi hospitals for better managing critically ill children at highest mortality risk.

## Background

Emergency care is among the weakest parts of health systems in low-income countries [1–5]. Sub-Saharan African facilities experience higher patient loads and mortality than other regions particularly for paediatric emergency patients [6]. Quality care is often impeded by adverse case management factors and emergency care is frequently poorly organised and lacking essential supplies [2]. Evidence suggests the quality of emergency care can be improved without huge investments if it is better organised and staff are trained to handle emergency situations in structured ways [7].

At the same time, lower-level facilities may have particularly limited possibilities to manage critically ill patients and must rely on adequate capacity to refer patients to hospitals or higher-level care as required. Yet time-sensitive care is challenged by potential delays during initial care-seeking to first-level facilities compounded by referral delays, which adds to the poor prognosis of already very sick patients [8, 9]. A maximum of 2 h travel time to facilities providing emergency care has been proposed as an international goal for surgical emergencies [10]. A similar target-setting process for accessibility of basic paediatric emergency care is desirable.

During the 1990s, the World Health Organisation (WHO) developed the Emergency Triage Assessment and Treatment (ETAT) guidelines to support hospitals in resource-poor settings to care for critically ill children [11]. These guidelines were field tested across multiple countries in the late-1990s showing reduced in-hospital paediatric deaths [7]. An evaluation in one Malawi hospital identified lack of staff and inadequate anaemia/malaria treatment as main limitations in delivering care for critically ill children [12]. The main causes of death in that hospital were malaria and malaria-related illnesses, pneumonia and malnutrition. A more recent study from a separate Malawi hospital found that nearly half (43%) of paediatric deaths were among infants, [13] and leading causes were sepsis, lower respiratory infections, gastroenteritis, meningitis and malaria.

Some governments, including Kenya and Malawi, have recently updated ETAT guidelines to provide more detailed instructions on equipment and processes needed for implementation [14]. This extended version of ETAT is known as ETAT Plus (ETAT+), and includes a hospital audit tool. While ETAT was previously implemented in Malawi hospitals, the Malawi Ministry of Health is currently developing its own ETAT+ manual to strengthen quality emergency care across its health system. Yet, to date, there has been no comprehensive assessment of Malawi hospital capacity to implement ETAT+ nor an understanding of commonly lacking equipment or supplies that could pose particular barriers to future implementation. While other research found suboptimal accessibility to surgery and emergency care in sub-Saharan Africa, these studies were based on population distance to hospitals with no assessment of hospital readiness to provide such care [3–5]. In this study, we used a national facility census with comprehensive facility audits to analyse Malawi hospital readiness to care for critically ill children according to the ETAT+ hospital audit tool. We further estimated travel times to emergency-equipped hospitals from non-equipped facilities and relative to Malawi’s population distribution.

## Methods

### Study setting

Malawi is a low-income country in sub-Saharan Africa with approximately 18 million people in 2016 including three million children under 5 years. Malawi’s health system is primarily comprised of government-run facilities and publicly-supported facilities run by the Christian Health Association of Malawi (CHAM). There are three main health system tiers: central hospitals, district hospitals, and peripheral facilities that include health centres, clinics, posts, maternities and dispensaries in our analysis. These facilities typically provide basic essential services including family planning, antenatal services, and outpatient care. The next level is the district hospital that are referral facilities providing inpatient care, laboratory diagnostics, and maternity wards. The highest level are central hospitals that are teaching and research centres with specialized medical services. To date, Malawi has targeted ETAT implementation to hospitals but going forward ETAT+ implementation will target both hospitals and health centres.

### Survey methods

The Malawi Service Provision Assessment (SPA) was conducted in June 2013–February 2014 by the Ministry of Health and The Demographic and Health Survey (DHS) Program, which includes facility audits, observed consultations, patient exit interviews and health worker interviews. All audited facilities were geocoded to allow for spatial analyses. Survey methods are described elsewhere including procedures for obtaining ethical approval and participant consent [15]. Briefly, Malawi SPA 2013–2014 was designed as a census of all formal public and private facilities in the country, which makes it distinct from other facility-based surveys to allow for the current investigation. An inventory questionnaire assessed facility readiness on the interview date to provide various services including: infrastructure, resources, and systems; maternal, new born, and child health; family planning; HIV/AIDS, malaria, and tuberculosis; minor surgery; and non-communicable diseases. There was no specific audit of emergency departments that may be found in larger hospitals with divided organisations of care, nor were there questions about emergency care organisation, training or triage practices. All audited facilities were included in this research.

### Emergency-equipped definition

The emergency-equipped definition in this study was based on components specified in the latest available ETAT+ hospital audit tool from the Kenyan Ministry of Health (Table 1) [14]. We assessed emergency readiness among hospitals since those facilities have been targeted for ETAT implementation to date. A hospital was considered emergency-equipped if all items were observed or reported available on the interview date. This definition reflects staff, transport, equipment, diagnostics, medications, fluids, feeds and consumables that underpin care for critically ill children. It does not include training, management or organisation of emergency care that are also important but were not assessed during the audit. Any component with a missing value was considered unavailable.

**Table 1:** Components of the study definition for emergency-equipped hospital

ETAT+ hospital audit components (14)	Emergency-equipped study definition
**STAFF**	Healthcare worker present or officially on-call 24 h per day
**TRANSPORT**	Functional ambulance or other emergency transport for clients
**EQUIPMENT**	
Weighing scale for infants	Infant weighing scale (100 g graduation)
Weighing scale for children	Child weighing scale (250 g graduation)
Oxygen source without sharing individual meters	Oxygen concentrator or filled oxygen cylinder
Complete suction equipment	Suction with catheter or bulb for newborns
Resuscitation equipment – BVM for all ages, NGT, needles and syringes	Self-inflating bag/mask for adult, child or neonate (see “Consumables” for other related equipment)
Nebulizer or spacer and mask for asthma treatment	Micro nebulizer or spacer for inhalers
Phototherapy equipment	Not audited
Infant warming devices	Not audited
**LABORATORY SERVICES**	
Blood slide microscopy or malaria RDT available	Malaria RDT or microscopy (giemsa stain, field stain or acridine orange)
Haemoglobin / haematocrit measurement	Haematology analyser or HemoCue equipment including microcuvettes or colorimeter/hemoglobinometer including Drabkins solution or litmus paper method for haemoglobin test services
Blood glucose measurement	Glucometer with test strips or blood chemistry analyser
Cross match and blood bank	Blood typing: anti-A, B, D and Coombs reagent
CSF microscopy and gram stain	Not audited
Radiology CXR	Digital x-ray with no film required, x-ray machine with unexpired film, or CT scan
**MEDICATIONS**	
1. **RESUSCITATION / EMERGENCY MEDICINES**	
Adrenaline 1 in 1000	Injectable epinephrine / adrenaline
Diazepam	Injectable diazepam
Glucose (10% or 50% for preparing 10%)	Injectable glucose solution (10% or 50%)
Hydrocortisone injection	Injectable hydrocortisone
Salbutamol inhaled	Inhalation salbutamol
IV salbutamol	Not audited
2. **ANTIBIOTICS**	Injectable antibiotics, any type
3. **ANTIMALARIALS**	Injectable antimalarials, any type
**FLUIDS**	
Intravenous fluids	Normal saline/sodium chloride injectable solution or dextrose 5%-normal saline or half-strength Darrow’s solution or Ringers lactate or IV solution (plasma expanders) with infusion set
**FEEDS**	
Feeds (e.g. F75, F100, formula feeds)	Not audited
**CONSUMABLES**	
Syringes 1, 2, 5, 10 & 20 mls	Single use standard disposable syringes with needles or auto-destruct syringes with needles
Blood transfusion sets	Blood transfusion services provided in past 3 months (yes/no)
Cannula / scale vein sets 18, 20, 22 & 24G	Cannula for administering IV fluids (21-24G)
Needles 18, 20, 22 & 24G	Listed above for “Syringes”
Suction catheters 8, 10 & 12G	Listed above for “Complete suction equipment”
Nasogastric tubes 6, 8, 10 & 12	Nasogastric tubes (10-16G)
Topical antiseptic for burns	Not audited
Sterile dressings	Not audited

### Data analysis

Geocoded facility locations were used to create maps characterizing travel times to any facility and emergency-equipped hospital based on methods from Weiss et al. 2018 [16]. Briefly, this approach applies a least cost path algorithm to facility points geolocated with a friction surface (e.g. 2D grid wherein each cell value is an estimate of the time it takes to move one meter within that cell). The friction surface accounts for surface travel through transportation networks (e.g. roads, railroads, and navigable waterways) and overland by identifying the fastest route between any two geolocated points. The road network datasets used in this analysis combined data from OpenStreetMap and Google, which creates the largest and most complete global road network datasets available.

There are two key assumptions of the travel time model. First, individuals will always travel by the fastest transport means, such as a vehicle on a road rather than walking. Second, travel times are static and do not account for elements such as traffic congestion, public transit delays or infrastructure changes (e.g. flooded roads). Based on these assumptions and available datasets, maps were produced such that each pixel value refers to the number of minutes required to reach the closest facility. We tabulated travel times from every non-equipped facility to its nearest emergency-equipped hospital as well as relative to population distributions by applying gridded population data from the WorldPop project to the travel time maps [17, 18].

National point estimates were tabulated using weights to account for unequal probabilities of selection due to facility non-response in this national facility census. We used Pearson’s chi-squared tests to determine whether median travel times to emergency-equipped hospitals differed across sub-national regions or rural/urban areas. The level of statistical significance was set to 0.05. Stata 13.1 (Stata Corp., College Station, TX) was used for this analysis.

## Results

The Malawi SPA 2013–2014 included 977 facilities of 1060 on the national facility list with non-response due to refusal, closure, inaccessibility or other issue (Table 2). Among these 977 facilities, 116 (12%) were hospitals while 861 (88%) were lower-level facilities including health centres, maternities, dispensaries, clinics, or health posts. 478 (49%) were government-run facilities, 160 (16%) were CHAM and 339 (35%) were managed by other authorities such as non-governmental organisations or private companies. A total of 167 (17%) facilities including 23 hospitals were located in the North region where approximately 2.2 million people resided in 2016; 364 (37%) facilities including 43 hospitals were in the Central region with approximately 7.3 million people; and 446 (46%) facilities including 50 hospitals were in the South region with about 7.8 million people.

**Table 2:** Characteristics of emergency-equipped hospitals in Malawi, 2013–2014

		Total facilities	Total hospitals	Emergency-equipped hospitals ^**a**^	
		**N**	**N**	**N**	**% (95% CI)**
**Total**	**Total**	**977**	**116**	**4**	**3.5 (1.3–8.9)**
Region	Central	364	43	1	2.3 (1.2–16.8)
	North	167	23	2	8.7 (0.6–25.2)
	South	446	50	1	2.0 (0.3–13.1)
Urban/Rural	Urban	299	62	2	3.2 (0.8–11.9)
	Rural	678	54	2	3.7 (0.9–13.8)
Managing authority	Government	478	50	1	2.0 (0.3–12.9)
	CHAM	160	41	3	7.3 (2.4–20.5)
	Other private	339	25	0	–

^a^There were 4 emergency-equipped facilities in Malawi including 3 hospitals and 1 health centre. Proportions of emergency-equipped facilities were tabulated among hospitals since those are targeted for ETAT implementation, although the future ETAT+ programme will be targeted to hospitals and health centres. Point estimates were also weighted to account for unequal probabilities of selection due to non-response. There were 1060 facilities on the national facility list and 977 (92.1%) facilities were audited in this national facility census. The level of statistical significance was set to 0.05

### Emergency-equipped facilities

**Fig. 1:**
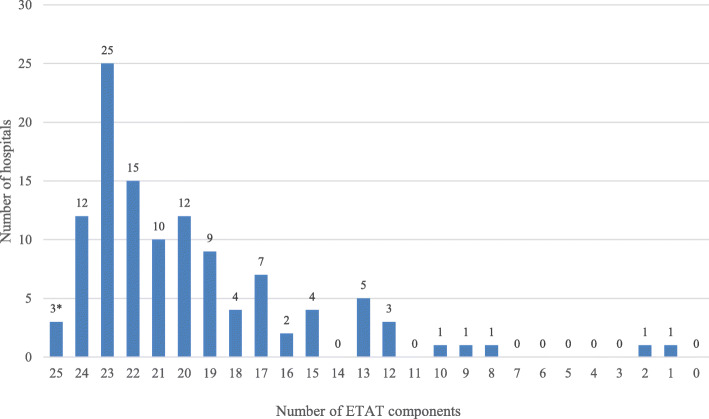
Distribution of Malawi hospitals by number of ETAT+ components observed or reported available on the audit date, 2013-2014. Note that three hospitals (and one health centre) had all 25 components specified in ETAT+ guidelines to fulfil the criteria for an emergency-equipped hospital

Four (3.5%; 95% CI: 1.3–8.9) Malawi facilities had all 25 components available on the interview date to fulfil study criteria for an emergency-equipped facility (Table 2). Among these four emergency-equipped facilities, three were hospitals and one was a health centre despite current ETAT implementation targeted only to hospitals. One was in the Central region while two and one were in the North and South, respectively. Two were in rural areas and two in urban districts. One government and three CHAM facilities were emergency-equipped. Figure 1 presents the distribution of Malawi hospitals according to the number of ETAT+ items observed or reported available on the audit date. While only 3 hospitals (plus one health centre) had all 25 items available to fulfil the emergency-equipped criteria, 74 (of 116 hospitals) had between 20 and 24 items available.

Table 3 shows availability of each component of the emergency-equipped definition among hospitals and all facilities. Least available items were nasogastric tubes in 34.5% (95% CI: 26.4–43.6) of hospitals followed by blood typing services (40.4, 95% CI: 31.9–49.6). Injectable hydrocortisone, micro nebulizers or spacers for inhalers, and radiology were found in about half of Malawi hospitals. Across all facilities, 33.0% (95% CI: 30.1–36.0) had a functional ambulance to transport critically ill patients as required.

**Table 3:** Availability of ETAT+ components in Malawi hospitals and lower-level facilities, 2013-2014

Category	Item	All facilities		Hospitals		Lower-level facilities	
		N	% (95% CI)	N	% (95% CI)	N	% (95% CI)
**Total**	**Total**	**977**	**100.0**	**116**	**100.0**	**861**	**100.0**
**Staff**	24-h staff on call	389	39.8 (36.7–42.9)	102	90.4 (83.4–94.6)	287	33.2 (30.1–36.4)
**Transport**	Ambulance	322	33.0 (30.1–36.0)	95	84.3 (76.5–89.9)	227	26.3 (23.4–29.3)
**Equipment**	Infant scale	692	70.8 (67.8–73.6)	105	92.9 (86.4–96.4)	587	67.9 (64.7–71.0)
	Child scale	757	77.5 (74.8–80.1)	98	87.0 (79.5–92.0)	659	76.3 (73.3–79.0)
	Oxygen source	239	24.4 (21.9–27.2)	102	90.4 (83.4–94.6)	137	15.8 (13.6–18.4)
	Suction with catheter/bulb for newborns	500	51.2 (48.0–54.3)	95	84.1 (76.2–89.7)	405	46.9 (43.6–50.2)
	Self-inflating bag/mask	622	63.6 (60.5–66.6)	104	92.1 (85.4–95.8)	518	59.9 (56.6–63.2)
	Micro nebulizer or spacer for inhalers	183	18.7 (16.4–21.3)	57	50.9 (41.9–60.0)	125	14.5 (12.3–17.0)
**Laboratory**	Malaria testing	853	87.3 (85.0–89.3)	109	96.5 (91.0–98.7)	745	86.2 (83.6–88.4)
	Haemoglobin measurement	202	20.6 (18.2–23.3)	91	81.0 (72.8–87.1)	110	12.8 (10.7–15.2)
	Blood glucose measurement	225	23.0 (20.5–25.7)	84	74.9 (66.2–82.0)	140	16.2 (13.9–18.9)
	Blood typing	57	5.9 (4.5–7.4)	46	40.4 (31.9–49.6)	11	1.3 (0.7–2.3)
	Radiology	69	7.1 (5.6–8.8)	61	54.2 (45.1–63.0)	8	0.9 (0.5–1.8)
**Medications**	Injectable epinephrine / adrenaline	606	62.1 (58.9–65.1)	91	80.9 (72.6–87.1)	515	59.6 (56.3–62.9)
	Injectable diazepam	776	79.4 (76.7–81.9)	106	93.9 (87.7–97.1)	670	77.5 (74.5–80.2)
	Injectable glucose solution	755	77.3 (74.5–79.8)	103	91.3 (84.5–95.3)	652	75.4 (72.4–78.2)
	Injectable hydrocortisone	194	19.9 (17.5–22.5)	55	49.2 (40.2–58.2)	139	16.1 (13.7–18.7)
	Inhalation salbutamol	323	33.0 (30.1–36.0)	76	67.3 (58.2–75.2)	247	28.5 (25.6–31.7)
	Injectable antibiotics	924	94.5 (92.8–95.8)	111	98.2 (93.2–99.6)	813	94.1 (92.2–95.5)
	Injectable antimalarials	859	87.9 (85.6–89.8)	106	93.8 (87.6–97.0)	753	87.1 (84.6–89.2)
**Fluids**	Intravenous fluids	829	84.8 (82.4–87.0)	109	96.4 (90.9–98.7)	720	83.3 (80.6–85.7)
**Consumables**	Syringes with needles	967	98.9 (98.0–99.4)	112	99.1 (93.9–99.9)	855	98.9 (97.9–99.4)
	Blood transfusion in past 3 months	73	7.4 (6.0–9.2)	70	61.8 (52.6–70.2)	3	0.3 (0.1–1.1)
	Cannula for administering IV fluids	815	83.4 (80.9–85.7)	110	97.4 (92.1–99.2)	706	81.6 (78.8–84.1)
	Nasogastric tubes	105	10.8 (9.0–12.9)	39	34.5 (26.4–43.6)	66	7.7 (6.1–9.7)

Point estimates were weighted to account for unequal probabilities of selection due to facility non-response. There were 1060 facilities included in the Ministry of Health national facility list and 977 (92.1%) facilities were audited as part of this national facility census

### Estimated travel times between facilities

Among the 973 non-equipped facilities, the median travel time was 73 min (95% CI: 67–77 min) to the nearest emergency-equipped hospital with a range of 1–507 min. In the North, the median travel time was 77 min (95% CI: 67–86 min), and in the South, the median travel time was 80 min (95% CI: 76–87 min). The median travel time in the Central region was 60 min (95% CI: 54–65 min), which was significantly lower than both other regions (*p* < 0.001). In urban areas, the median travel time was 44 min (95% CI: 42–46 min) to the nearest emergency-equipped facility, which was significantly lower than in rural areas with a median travel time of 87 min (95% CI: 82–90 min) (*p* < 0.001).

### Estimated travel times by population distributions

**Fig. 2:**
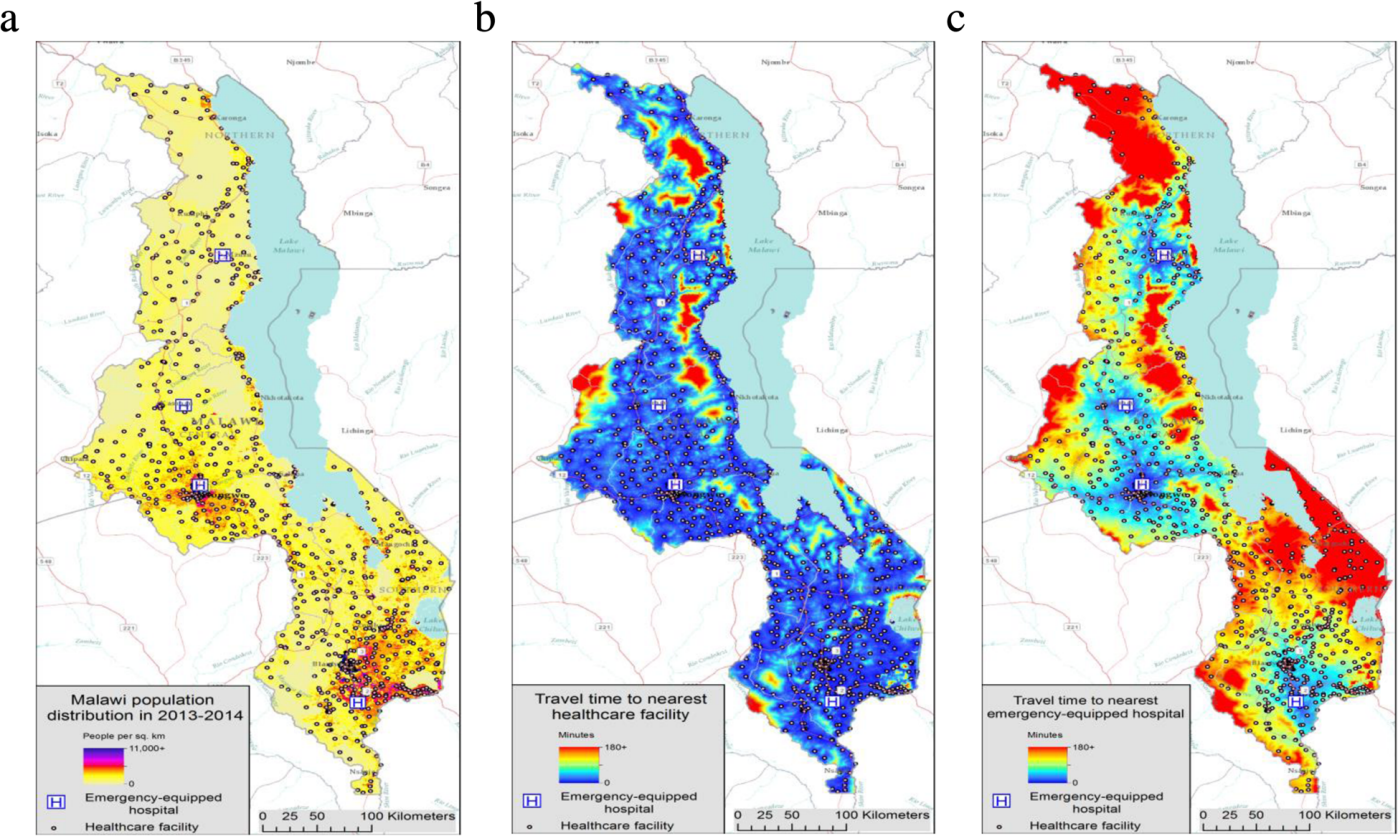
Malawi population distribution and travel times to the nearest health facility and emergency-equipped hospital, 2013-2014. **a** Map 1: Malawi population distribution in 2013-2014; **b** Map 2: Estimated population travel time (minutes) to the nearest health care facility in Malawi in 2013-2014; **c** Map 3: Estimated population travel time (minutes) to the nearest emergency-equipped hospital in Malawi in 2013-2014. Maps for this study were produced by Malaria Atlas Project, University of Oxford. The World Reference Overlay (data sources: ESRI, Garmin, USGS, NPS) was used as the base map

**Fig. 3:**
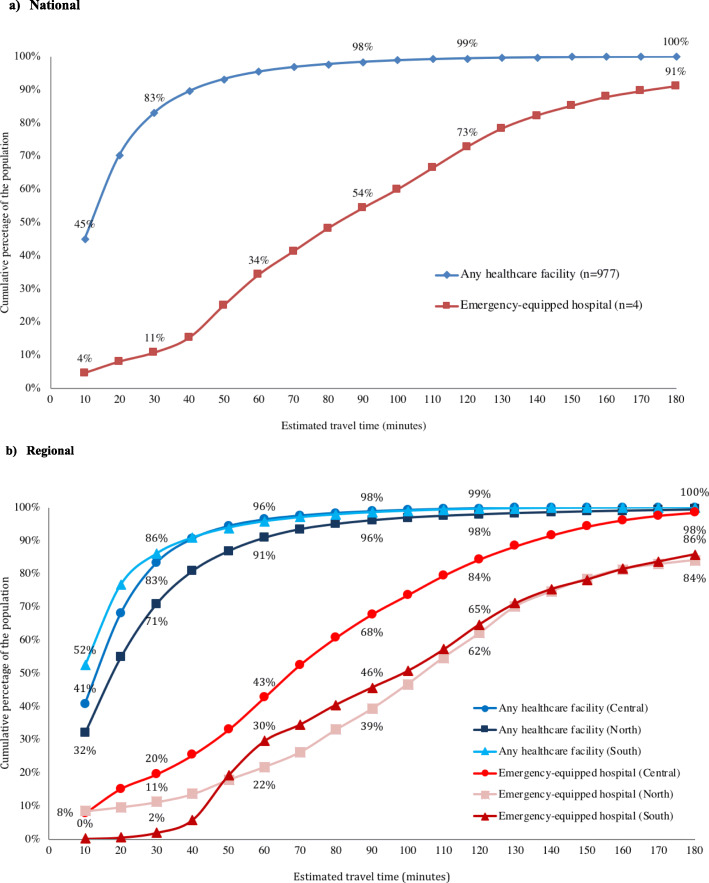
Proportional distribution of population travel times to the nearest health facility and emergency-equipped hospital, 2013-2014. Figure (**a**) National; Figure (**b**) Regional

We estimated that 45% of the Malawi population had no more than a 10-min travel time to any facility while 83 and 95% of the population lived within a 30- and 60-min travel time to any facility, respectively (Fig. 2). In contrast, only 4% of the Malawi population lived within a 10-min travel time of an emergency-equipped facility while 11% lived within 30-min travel time and approximately one-third (34%) of the population lived within 60 min. More than one-quarter (27%) of Malawi’s population, or approximately 4.7 million people, must travel over 120 min to an emergency-equipped hospital. There were also significant regional differences in estimated travel times to an emergency-equipped hospital (Fig. 3). The percentage of the population that must travel over 120 min to an emergency-equipped hospital was 16% in the Central region compared to 35 and 38% in the South and North regions, respectively (*p* < 0.001).

## Discussion

Overall, only four Malawi hospitals were fully equipped to provide basic paediatric emergency care according to the study definition. More than one-quarter (27%) of Malawi’s population – or approximately 4.7 million people – must travel more than 120 min to reach an emergency-equipped hospital with significant regional differences in travel times.

Our findings are more pessimistic than previous studies that estimated only 7% of Malawi’s population live more than 2 h from a public hospital with emergency care [5]. Another recent study found that 92.5% of the sub-Saharan African population lived within 2 h of a major hospital for surgical procedures [3]. However, actual services provided by each hospital were not known in the aforementioned studies and those results likely overestimate health system capacity to provide emergency care. The current study is the first to our knowledge to map travel times to emergency care for critically ill children in a low-income country by linking a national facility census that combined comprehensive inventory audits with global road network datasets to more accurately estimate population accessibility both nationally and at sub-national levels.

Insufficiencies of equipment and supplies for emergency care within hospitals in sub-Saharan Africa is a well-described problem, [2, 19] and constitutes a major barrier to the provision of quality care and adherence to international guidelines [20]. Lack of basic equipment with associated failures to provide the care needed may create a vicious cycle whereby patients’ experienced and perceived poor quality care at healthcare settings can further contribute to treatment failures [21]. A hospital’s ability to provide the care required by visiting clients is important since it may lead to improved and more timely utilization of services and thus better patient outcomes.

While our study showed great improvements in hospital readiness to manage severe malaria compared to previous ETAT evaluations in Malawi, [13] other deficiencies were found in these results. Specifically, least available items were nasogastric tubes, blood typing services, injectable hydrocortisone, micro nebulizers or spacer inhalers and radiology. These deficiencies are especially disconcerting considering that low- and middle-income countries bear the greatest burden of death from lung disease, [22] and paediatric anaemia [23]. Insufficiencies in the health infrastructure reduces both quantity and safety of blood supplies in low- and middle-income countries, [24] and improved capacity to provide blood transfusions would be life-saving since mortality from anaemia remains high [25]. Our study also found nasogastric tubes commonly lacking in hospitals, which may pose a barrier to delivering quality care for dehydrated and malnourished children. However, this result could partly reflect data collection issues since only specific sizes of nasogastric tubes were audited with common child sizes (6 and 8G) not assessed.

Paediatric asthma is commonly underdiagnosed in low-income settings with associated high mortality rates, [26] and most Malawi facilities lack equipment and medications needed to manage an asthma exacerbation. While oxygen was commonly available in Malawi hospitals, only 15% of lower-level facilities reported oxygen availability despite its inclusion in the WHO essential medicines list [27]. The cost effectiveness of an oxygen system strategy has been shown to compare favourably with other child survival interventions [28]. An implementation effectiveness trial of sustainable and renewable oxygen and power systems in remote areas of low-income countries is underway and could provide promising results [29].

The recent WHO report on quality of care recommends timely referral for every child with conditions that cannot be managed effectively at first-level facilities [30]. This is an obvious challenge in the Malawi health system where only one-third of facilities have a functional ambulance. Minimum standards for paediatric emergency care in remote and resource-poor settings are not well-defined but pre-referral management using simple equipment and supplies could improve survival chances of very sick patients [31]. Recent studies show that improved pre-hospital care was achieved by training commercial taxi/minibus drivers to provide basic emergency care [32]. Implementation of motorcycle ambulances have also reduced referral times for obstetric care in Malawi [33]. Other innovations to address referral challenges should be explored.

While our results indicate that 73% of Malawi’s population live within 120 min travel time to an emergency-equipped hospital, this apparently high figure must be considered in light of related issues. First, there are significant in-country regional differences in access to basic paediatric emergency care with worse population accessibility in North and South than Central regions. Similar inequities have been demonstrated in paediatric pneumonia assessment practises [34]. Second, the purpose of emergency care is to provide urgent medical interventions for time-critical health problems making prompt care essential for entire populations. Third, while travel times are estimated based on road networks, other barriers likely impede travel to hospitals such as financial and physical availability of transport and additional difficulties of transporting severely sick children [35]. Malawi roads may also not be fully developed or there could be other road difficulties that further reduce travel times or require people to walk rather than using other transport means. It is thus expected that actual travel times are longer than ones presented here. Indeed, while travel time are useful in a relative sense (e.g. distinguishing highly accessible areas from remote ones) they provide best-case-scenario values that cannot be considered universally applicable.

Malawi has achieved impressive reductions in child mortality and achieved Millennium Development Goal Four (MDG4) by 2013 [36]. This progress has mainly been explained by high and equitable coverage with high-impact preventive interventions including malaria bed net distribution and reductions in malnutrition. While preventive efforts must be sustained, further decreases in child mortality in Malawi and other low-income countries will require substantial investments to expand emergency care to better manage critically ill children at highest mortality risk.

### Methodological limitations

There are a number of methodological considerations in this study. First, equipment and supplies were assessed through audits of general outpatient departments and other service delivery sites. SPA did not specifically audit emergency departments although minor surgery sites were assessed in every facility. This should mainly affect larger hospitals more likely to have a divided organisation of care. Training, management and organisation of emergency care within each facility were not assessed, nor were quality of care or service utilization outcomes. Second, the emergency-equipped definition included items that were either observed or reported available as well as either functioning or not functioning/don’t know on the interview date. This definition may misclassify some facilities as emergency-equipped if items were reported available/functioning but were not. Third, only Malawi facilities were included in the analysis and some populations may have shorter travel times to facilities in neighbouring countries. Fourth, emergencies requiring surgical and/or orthopaedic interventions were not included and involve additional management challenges that should be considered in developing emergency care capacity of a health system. Fifth, data were collected during 2013–2014 and may not reflect current ETAT+ readiness in Malawi hospitals. However, it is not expected that paediatric emergency medicine has substantially improved since this time given the lack of major sector-specific investments. Finally, and as previously discussed, travel time estimates are best-case scenario values that indicate relative distances to emergency care for different populations and geographic areas within Malawi. These estimates are not universally applicable such that individual access to emergency care depends on use (or not) of motorised transport, as one example, that would greatly facilitate or impede individual travel times to emergency care.

## Conclusions

Based on a national census of 977 facilities in Malawi in 2013–2014, study findings indicate severely limited accessibility to hospitals equipped to manage critically ill children with particularly deficient capacity to treat childhood anaemia and respiratory illnesses. Population access to emergency-equipped hospitals was unevenly distributed across regions and urban/rural areas that should be considered in future health system planning and investments. There is an urgent need to strengthen Malawi health system capacity to manage basic paediatric emergencies including reliable supply chains for essential drugs and commodities and time-sensitive referral for transporting patients to higher-level care as required. While Malawi and other low-income countries have made significant progress in reducing child mortality over the past few decades, further gains will require substantial investments to expand quality emergency care to improve survival chances of critically ill children at highest mortality risk.

## Data Availability

The data that support the findings of this study are publicly available from The DHS Program at https://dhsprogram.com.

## Abbreviations

ETAT: Emergency Triage, Assessment and Treatment
MDG: Millennium Development Goal
SPA: Service Provision Assessment
WHO: World Health Organisation
